# DCE-MRI reveals spatial pattern in heterogeneous blood-brain barrier leakage within white matter in cerebral small vessel disease

**DOI:** 10.1177/0271678X251364151

**Published:** 2025-08-04

**Authors:** Damon Verstappen, Joost J.A. de Jong, Paulien H.M Voorter, Maud van Dinther, Robert J van Oostenbrugge, Julie Staals, Jacobus F.A Jansen, Walter H Backes

**Affiliations:** 1 Department of Radiology & Nuclear Medicine, Maastricht University Medical Centre+, Maastricht, Netherlands; 2 Mental Health and Neuroscience Research Institute (MHeNs), 5211 Maastricht University, Maastricht, Netherlands; 3 Department of Neurology, Maastricht University Medical Centre+, Maastricht, Netherlands; 4 Cardiovascular Research Institute Maastricht (CARIM), 5211 Maastricht University, Maastricht, Netherlands; 5 Department of Electrical Engineering, 3169 Eindhoven University of Technology, Eindhoven, Netherlands

**Keywords:** Blood-brain barrier, Cerebral small vessel disease, Dynamic contrast-enhanced MRI, Vascular cognitive impairment, White matter hyperintensities

## Abstract

Cerebral small vessel disease (cSVD) is associated with vascular cognitive impairment, dementia, and stroke. Blood-brain barrier (BBB) dysfunction is central to its pathophysiology and is involved in the formation of tissue lesions. The spatial heterogeneity of BBB leakage remains largely unclear. This cross-sectional study assessed BBB leakage rate (K_i_), fractional volume of leaking tissue (v_l_), and blood plasma volume (v_p_) in various tissue regions, including gray matter (GM), normal appearing white matter (NAWM), and white matter hyperintensities (WMH) of 59 patients with cSVD and 32 controls using a high spatial resolution dynamic-contrast enhanced MRI protocol. Using regionally averaged measures, patients with cSVD had higher v_l_ (p = 0.020) and lower v_p_ (p < 0.001) within WMH compared to controls, K_i_ did not differ in any region. To evaluate the spatial heterogeneity of leakage in the NAWM, we analyzed 2-mm-wide shells extending outward from WMH edges. This revealed stronger BBB leakage in perilesional NAWM (p = 0.032) of cSVD patients compared to controls, with a striking dip close to the WMH. K_i_, v_l_, and v_p_ increased with distance from WMH edges (all p < 0.001). This pattern of lower BBB leakage in the perilesional NAWM could be caused by local reductions in microvascular blood flow or vessel surface area.

## Introduction

Cerebral small vessel disease (cSVD) is a term to describe pathologies that affect the brain microvasculature, which is associated with vascular cognitive impairment (VCI), dementia, and stroke.^1,2^ One of the key imaging biomarkers of cSVD is the presence of white matter hyperintensities (WMH).^3^ These WMH are presumed to occur due to underlying microvascular blood-brain barrier disruption and tend to expand over time into the surrounding normal appearing white matter (NAWM).^3,4^ In order to better understand the progression, research into the condition of the perilesional ‘at risk’ tissue, which we know to be affected by hypoperfusion and fluid accumulation, is warranted.^5–7^ Furthermore, both magnetic resonance imaging (MRI) and histological studies have shown that WMH and surrounding NAWM tissue characteristics vary with location.^5,8,9^

The MRI-based pharmacokinetic measure for BBB leakage rate K_i_ is not only governed by BBB permeability, but also by vessel surface area and blood flow. In cSVD, these can be affected by microvascular alterations such as rarefaction and hypoperfusion.^10^ These factors may vary spatially in a way that masks or amplifies underlying leakage changes. Consequently, conflicting reports exist regarding the spatial heterogeneity of BBB leakage in the NAWM closest to WMH, with some studies finding a decreasing BBB leakage gradient radiating out from WMH^11,12^, while others do not.^5,13^ Additionally, focal ‘hotspots’ of increased BBB permeability have been observed within white matter lesions and their surroundings.^14^ This calls into question the assumption that the NAWM can be assessed as a single homogeneous tissue region in dynamic contrast-enhanced (DCE) MRI studies for assessment of BBB leakage.

As the BBB leakage in cSVD is subtle, the K_i_ maps appear rather noisy, hindering detailed spatial analysis. For this reason, we utilized a special DCE MRI protocol with increased sensitivity to low contrast agent concentrations and 1-mm spatial detail. Using this protocol, we aimed to determine how the BBB leakage was different in the various tissue regions in cSVD and control subjects, as well as how it varied in NAWM with distance from WMH.

## Methods

### Study population

This work includes data acquired for the CRUCIAL-VCI (miCRovascular rarefaction in vascUlar Cognitive Impairment and heArt faiLure) study (Trial Registration: ISRCTN22301128).^15^ For this work, we focus on the subset of patients with VCI due to cSVD. Patients with clinically overt cSVD were recruited between December 2020 and October 2024 at the Maastricht University Medical Centre+, the Netherlands. These patients were diagnosed with VCI and/or lacunar infarction and were recruited from the local memory or stroke clinic. VCI due to cSVD was defined as: (1) subjective complaints of cognitive functioning, (2) objective cognitive impairment in at least one cognitive domain in neuropsychological assessment or a Montreal Cognitive Assessment (MoCA) score <26, and (3) imaging evidence of cSVD^16^, defined as extensive leukoaraiosis on CT, or moderate to severe WMH on MRI (Fazekas score ≥2), or mild WMH (Fazekas score = 1) in combination with lacunes or microbleeds.^3^ Patients with a Clinical Dementia Rating score >1 were excluded.^17^ Lacunar stroke (due to cSVD) was defined as: a classical lacunar stroke syndrome with a compatible lesion on CT or MRI, and additional imaging evidence of cSVD (mild to severe WMH and/or microbleeds). To minimize the effects of acute stroke, lacunar stroke patients were enrolled at least 3 months post-stroke. We additionally recruited sex- and age matched controls from the neurology outpatient clinic where they presented with minor compression mononeuropathy or lumboischialgia. These controls had no cognitive impairment or overt cerebrovascular disease. Covert signs of SVD, like minor WMH, were not ruled out before inclusion.

Exclusion criteria common to both cSVD patients and controls comprised other neurological or psychiatric conditions affecting the brain and general contra-indications to be able to undergo MRI or tolerate gadolinium-based contrast agents. This study was approved by the Medical Ethics committee of the Maastricht University Medical Centre (approval number: NL72696.068.20). All participants provided written informed consent in accordance with the declaration of Helsinki.

### MRI

All images were acquired using a 3 T MRI system (Ingenia CX, Philips Healthcare, Best, the Netherlands), using a 32-element head coil. The DCE-MRI protocol consisted of multiple time phases, which started with the acquisition of a pre-contrast T1 map using a double inversion-time (TI) gradient echo (GE) sequence with the following parameters: TR = 8.3 ms, TE = 3.8 ms, TI_1_ = 650 ms, TI_2_ = 2100 ms, FA = 6°, matrix size: 240 × 240 with 1 mm cubic resolution, 180 slices and a total acquisition time of 4:36 (min:s). This was followed by a fast saturation recovery 3 D DCE scan (TR = 5.3 ms, TE = 1.6 ms, TD = 120 ms, FA = 30°, matrix size 128 × 128, voxel size: 2 × 2 × 6 mm, number of slices: 21, dynamic scan interval: 1.46 s, total acquisition time of 2:09 (min:s)) before and during contrast agent (0.1 mmol gadobutrol (Gadovist®) per kg body weight) bolus administration at an injection rate of 3 mL/s (Table A1). Immediately afterwards and 20 to 30 minutes post-contrast agent administration, the same T1 mapping sequence as the pre-contrast one was repeated.^18^ A T2-weighted FLAIR image with the same voxel size as the double TI gradient echo sequence was additionally acquired to aid WMH visualization.

### Image analysis

The images were automatically segmented using the software package SAMSEG into the following brain regions of interest (ROI): a combination of cortical and deep gray matter (GM), NAWM, and WMH.^19^ The resulting WMH segmentations were visually checked, and manually corrected if necessary. Any other abnormalities such as infarcts were manually segmented and removed from the tissue region masks.

The fast DCE images were motion corrected (*FSL mcflirt v6.0.4*^20^) and all images and masks were spatially coregistered (FSL *flirt v6.0.4*^20^) to the first post-contrast T1 map. The vascular input function (VIF) for pharmacokinetic modeling was obtained from a manually selected region in the superior sagittal sinus.^21^ For each patient, hematocrit levels from a recent venous blood sampling were used to correct the contrast agent concentration (C_plasma_ = C_blood_/(1 - hematocrit)), yielding the concentration in blood plasma. The graphical Patlak approach was used to calculate the contrast agent leakage rate (K_i_) and volume fraction of blood plasma (v_p_) per voxel.^22^ Subsequently the voxel-wise mean K_i_ (normally distributed) and median v_p_ (positively skewed) were calculated per tissue ROI. The approach of van de Haar, et al.^23^ was employed to obtain the fractional volume of leaking tissue (v_l_) for each ROI. In short, histograms of the K_i_ values were created per ROI. These histograms show positive, negative, and zero leakage values. The negative leakage values were used as an estimation of half of the noise and the full noise distribution was determined by mirroring this around K_i_ = 0. The full noise estimation was subsequently subtracted from the histogram. The remaining area under the curve of the positive side of the histogram represents the v_l_ of this tissue ROI.

**Figure 1.**
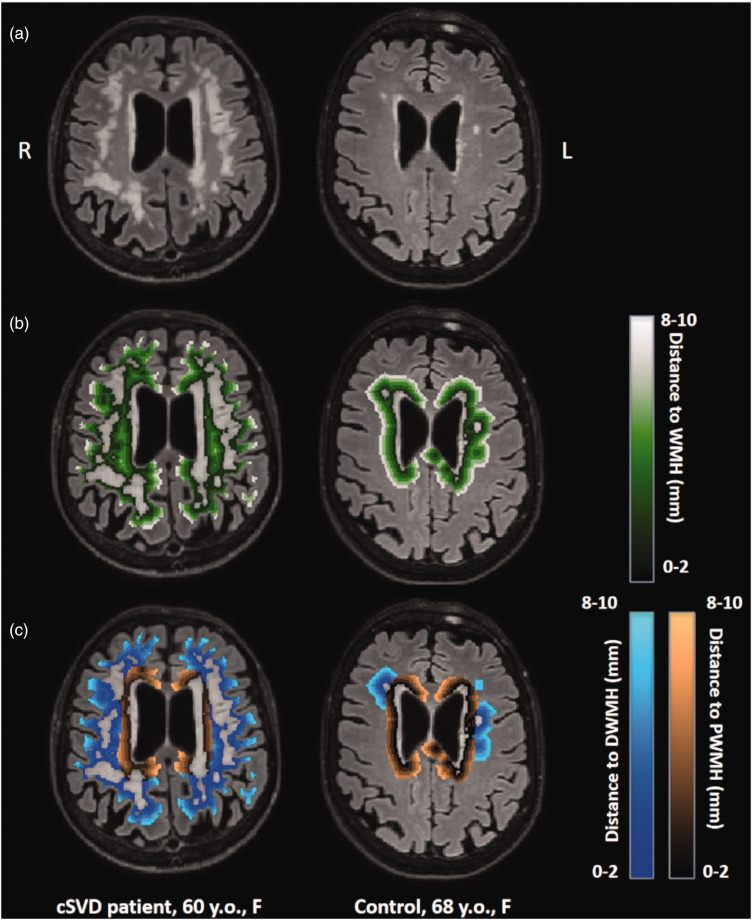
(a) T2-weighted FLAIR scans with white matter hyperintensities (WMH) for a 60-year-old female cSVD patient (left) and for a 68-year-old female control (right). (b) Visualization of 2-mm-thick shells around all WMH and (c) visualization of 2-mm-thick shells around the periventricular WMH (PWMH) and the deep WMH (DWMH).

For perilesional shell analysis, WMH were classified as either periventricular WMH (PWMH), i.e. all lesions within 10 mm distance from the ventricle edge, or deep WMH (DWMH), i.e. lesions >10 mm distance from ventricle edge.^24^ This definition has been shown to provide the best separation between WMH subtypes and correlates to microstructural, functional, and clinical measures.^25^ To allow for the evaluation of BBB leakage heterogeneity in the NAWM, for both the PWMH and DWMH, 5 shells, each 2 mm thick were defined from the edge of their respective WMH regions, overlapping with the NAWM contour. PWMH shells were confined to the region within 10 mm from the ventricles, while DWMH shells were confined outside that region. Additionally, five 2-mm-thick NAWM-overlapping shells were defined from the closest WMH, either periventricular or deep (Figure 1).

### Statistical analysis

All statistical analyses were performed in Python (v3.11.5), using the SciPy (v1.11.3) and statsmodels (v.0.14.0) libraries. The normal distribution of the data for each parameter was checked using the Kolmogorov-Smirnov test. Differences in demographic and clinical characteristics between groups were assessed using independent samples t-tests for normally distributed continuous data, Mann–Whitney U tests for non-normally distributed continuous data, and χ^2^ tests for categorical data. Differences between tissue types in terms of K_i_, v_l_, and v_p_ were first assessed using a one-way repeated-measures ANOVA for each variable. Subsequently, we used paired t-tests between each pair of GM, NAWM, and WMH to test which pairs differed significantly across all participants. K_i_, v_l_, and v_p_ in NAWM, WMH, and GM were compared between cSVD patients and controls using age- and sex-corrected multivariable linear regression. Paired samples t-tests were used to see if there was a difference in pharmacokinetic parameters between deep and periventricular WMH contours. Separate linear mixed-effect models were used to evaluate how these parameters varied over the shells surrounding PWMH, DWMH, and all WMH. Fixed effects included shell distance, patient/control group, age, and sex. A random intercept was included for each patient, assuming independent and identically distributed (i.i.d.) residuals, to account for individual variations. For sensitivity analyses, we further stratified the cSVD group by clinical presentation (VCI or lacunar stroke) and repeated the shell-based analyses within each subgroup. For all analyses, a p-value <0.05 was considered statistically significant.

## Results

Out of 99 participants recruited in the CRUCIAL study, eight were excluded from the analysis. Four participants discontinued scanning due to discomfort, two participants were lost due to data storage errors, one was excluded due to presence of traumatic brain lesions, and one control participant was excluded because cognitive impairment was present upon neuropsychological evaluation. The final cohort comprised 59 cSVD patients and 32 age- and sex-matched controls. Participant characteristics can be found in Table 1. Patients with cSVD had larger WMH volume compared to controls.

**Table 1.** Participant characteristics (N = 91).

	cSVD patients (n = 59)	Controls (n = 32)	p-value
Female sex, N (%)	18 (31%)	9 (28%)	1.000
Age (years), mean (SD)	70.0 (9.1)	69.2 (7.1)	0.657
Lacunar stroke/Vascular cognitive impairment (VCI), N (%)	16 (27%)/43 (73%)	N/A	**N/A**
Mini-Mental State Examination (MMSE), mean (SD)	27.1 (3.4)	28.8 (1.4)	**0.006**
WMH volume, normalized to intracranial volume, mean (SD)	0.017 (0.013)	0.0026 (0.0029)	**<0.001**
Total Fazekas score (median, IQR)	5 (3–6)	1 (0.5–2)	**<0.001**
WMH volume (cm^3^), mean (SD)	25.29 (19.77)	3.89 (4.18)	**<0.001**
Periventricular WMH volume (cm^3^), mean (SD)	15.55 (11.45)	2.77 (3.01)	**<0.001**
Deep WMH volume (cm^3^), mean (SD)	9.74 (10.03)	1.12 (1.96)	**<0.001**
Body mass index (BMI), mean (SD)	26.9 (4.2)	26.1 (3.2)	0.307
History of hypertension, N (%)	47 (80%)	18 (56%)	**0.034**
Systolic blood pressure (mmHg), mean (SD)	132.6 (17.3)	140.6 (19.9)	0.060
Diastolic blood pressure (mmHg), mean (SD)	85.5 (11.0)	87.8 (13.3)	0.409
Estimated glomerular filtration rate (eGFR) (mL/min), mean (SD)	73.2 (14.4)	71.0 (13.9)	0.477
Diabetes, N (%)	12 (20%)	5 (16%)	0.788
Smoking history, N (%)	37 (63%)	19 (59%)	0.931
Hypercholesterolemia, N (%)	50 (85%)	18 (56%)	**0.006**
History of ischemic stroke, N (%)	32 (54%)	0 (0%)	**N/A**
History of transient ischemic attack (TIA) N (%)	19 (32%)	3 (9%)	**0.030**

*N= number, IQR = interquartile range, SD = standard deviation, Total Fazekas score: the sum of deep Fazekas score (0–3) and periventricular Fazekas score (0–3)*.

### Pharmacokinetic parameters per tissue type

**Figure 2.**
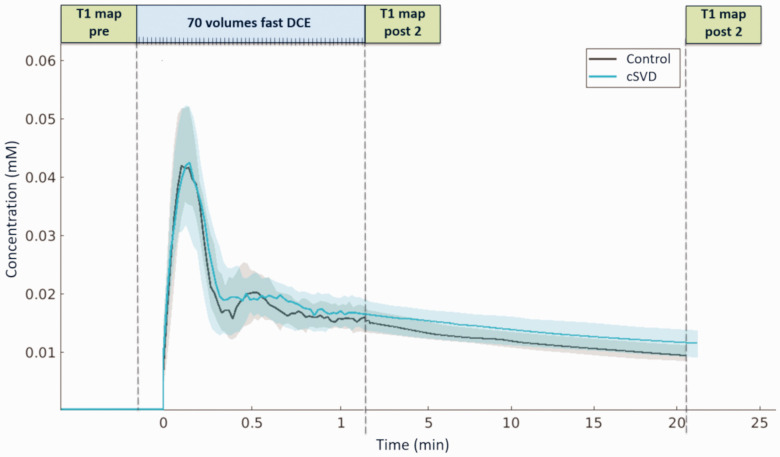
Sampling scheme and contrast agent concentration (median, IQR) over time across patients and controls in the shell 4–6 mm from the white matter hyperintensities (WMH).

Contrast agent concentrations over time for the example of a perilesional shell are visualized in Figure 2. Each tissue type pairing of GM, NAWM, and WMH differed significantly in terms of K_i_, v_l_, and v_p_, with the weakest leakage (both K_i_ and v_l_) in NAWM. Regionally averaged K_i_ did not differ between patients and controls in the GM, NAWM, and WMH. In WMH, v_l_ was significantly higher in patients (p = 0.020). Vp was significantly lower in patients than controls in the WMH (p < 0.001), but not in the GM or NAWM (Table 2).

**Table 2.** Pharmacokinetic measures. Leakage rate K_i_, v_l_, and v_p_ in the gray matter (GM), normal appearing white matter (NAWM), and white matter hyperintensities (WMH) and multivariable linear regression model results.

Measure	cSVD, mean (SD)	Control, mean (SD)	Covariate	Coefficient (SE)	P-Value
Leakage rate K_i_ (∙10^−3 ^min^−1^)					
GM	0.425 (0.123)	0.433 (0.158)	cSVD	−0.0100 (0.0289)	0.729
			Age	0.0039 (0.0017)	**0.020**
			Female sex	−0.0561 (0.0302)	0.066
NAWM	0.156 (0.105)	0.134 (0.111)	cSVD	0.0202 (0.0232)	0.385
			Age	0.0029 (0.0013)	**0.032**
			Female sex	−0.0214 (0.0242)	0.379
WMH	0.217 (0.130)	0.174 (0.235)	cSVD	0.0432 (0.0378)	0.255
			Age	0.0026 (0.0022)	0.237
			Female sex	−0.0668 (0.0395)	0.094
v_l_					
GM	0.357 (0.125)	0.368 (0.148)	cSVD	−0.0102 (0.0296)	0.731
			Age	−0.0010 (0.0017)	0.553
			Female sex	−0.0006 (0.0310)	0.984
NAWM	0.158 (0.107)	0.150 (0.104)	cSVD	0.0073 (0.0234)	0.756
			Age	0.0016 (0.0013)	0.238
			Female sex	−0.0152 (0.0244)	0.535
WMH	0.249 (0.138)	0.178 (0.123)	cSVD	0.0692 (0.0292)	**0.020**
			Age	0.0025 (0.0017)	0.142
			Female sex	−0.0095 (0.0305)	0.756
v_p_					
GM	0.0267 (0.0056)	0.0252 (0.0042)	cSVD	0.00135 (0.00110)	0.219
			Age	0.00017 (0.00006)	**0.010**
			Female sex	0.00172 (0.00115)	0.138
NAWM	0.0157 (0.0037)	0.0148 (0.0024)	cSVD	0.00073 (0.00069)	0.288
			Age	0.00009 (0.00004)	**0.023**
			Female sex	0.00152 (0.00072)	**0.037**
WMH	0.0122 (0.0031)	0.0150 (0.0045)	cSVD	−0.00279 (0.00081)	**<0.001**
			Age	−0.00001 (0.00005)	0.811
			Female sex	−0.00104 (0.00084)	0.223

### Associations of leakage parameters with age and sex

K_i_ showed a significant increase with age in the GM (p = 0.020) and NAWM (p = 0.031), while v_l_ was unrelated to age in each region. V_p_ increased with age in GM (p = 0.001) and NAWM (p = 0.023). K_i_ and v_l_ were not related to sex, but v_p_ was higher in females compared to males in the NAWM (p = 0.037) and unrelated to sex in the other regions (Table 2 and Figure A1).

### Deep versus perventricular WMH

Deep and periventricular WMH tissue differed in terms of v_l_ and v_p_, but not K_i_; v_l_ was higher (mean difference = 0.046, 95% CI [0.013 to 0.080], p = 0.007) and v_p_ was lower (mean difference = −0.0047, 95% CI [−0.0055 to −0.0039], p < 0.001) in DWMH compared to PWMH across all participants.

### Perilesional shell analysis

**Figure 3.**
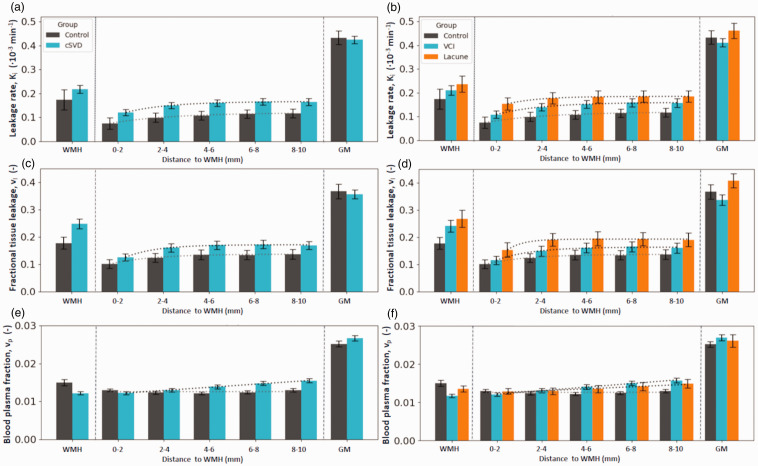
(a, b) Spatial leakage profile. K_i_ (mean, SE) and fit line in white matter hyperintensities (WMH), in the surrounding normal appearing white matter (NAWM) split in 2-millimetre-thick shells surrounding the WMH contours, and in the gray matter (GM). Note how the K_i_ plateaus from the 4-6-millimetre-shell. (c, d) Spatial distribution of leakage volume (v_l_) (mean, SE) and (e, f) spatial distribution of the blood plasma fraction (v_p_) (mean, SE). The panels on the right split up the cSVD group by inclusion criterion, either due to vascular cognitive impairment (VCI) or due to a lacunar infarction.

K_i_ in sequential perilesional shells of NAWM outside WMH was higher for more distant shells in patients compared to controls (p = 0.032) (Figure 3(a)). This effect was mainly driven by proximity to DWMH, as the model including shells drawn from the DWMH showed high significance (p = 0.005). No such relationship was found for PWMH (p = 0.125). No difference between patients and controls was found when evaluating v_l_ across shells. For patients compared to controls, v_p_ was higher in shells around DWMH (p = 0.032), whereas this relation only showed a trend for shells surrounding PWMH (p = 0.078). Effects of age and sex, as well as the full table can be found in the supplementary materials (Table A2).

### Distance effects from WMH boundary

**Figure 4.**
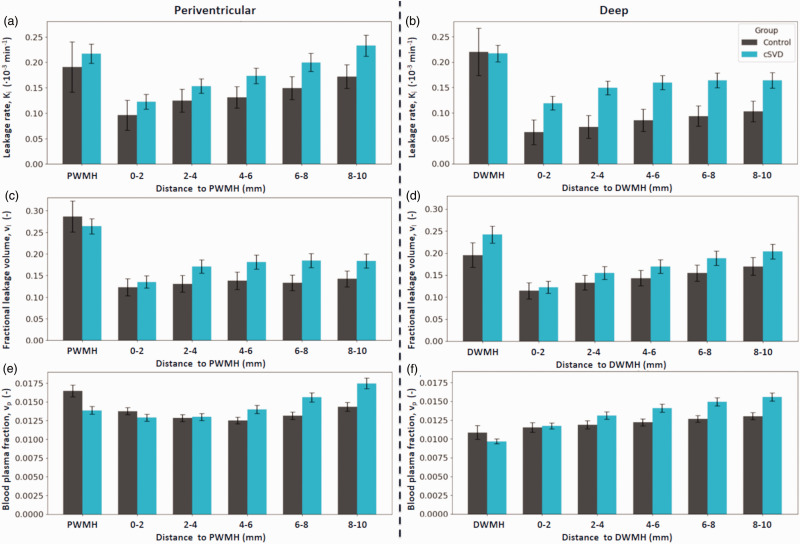
(a, b) Spatial leakage profile. K_i_ (mean, SE) in white matter hyperintensities (WMH), in the surrounding normal appearing white matter (NAWM) split in 2-millimetre-thick shells surrounding their respective WMH contours. (c, d) Spatial distribution of leakage volume (v_l_) (mean, SE) and (e, f) spatial distribution of the blood plasma fraction (v_p_) (mean, SE).

Evaluated across both groups, K_i_, v_l_, and v_p_ each increased significantly with increasing distance from WMH, both PWMH and DWMH, with p < 0.001. This is illustrated in Figure 3 for K_i_, v_l_, and v_p_ surrounding all WMH. Here, for both patients and controls, a plateau could visually be observed from the 4–6 mm shell for K_i_ and v_l_. Patients showed increasing v_p_ with distance from WMH, without plateauing, while the v_p_ for controls showed no distance effect. Graphs for PWMH and DWMH can be found in Figure 4.

### Sensitivity analyses by clinical presentation

Sensitivity analyses stratified by clinical presentation showed that lacunar stroke patients (n = 16) were younger (mean (SD): 61.6 (7.5) years) than VCI patients (n = 43, 73.1 (7.6) years, p < 0.001), had lower WMH volumes (14.8 (13.3) vs 29.2 (20.5) cm³, p = 0.003), and higher MMSE scores (29.0 (1.2) vs 26.3 (3.7), p < 0.001). When comparing both groups to controls, stronger BBB leakage in perilesional NAWM was most apparent in the lacunar stroke group (p < 0.001), while the K_i_ in the VCI group did not differ significantly in shells surrounding all WMH (p = 0.348). Distance-dependent effects from the WMH boundary were consistent with previous analyses (each p < 0.001, Figure 3). The regression results are described in Table A3.

## Discussion

This study investigated the spatial heterogeneity of BBB leakage in the perilesional NAWM in patients with cSVD and elderly controls. We showed that the BBB in the NAWM surrounding WMH leaks more strongly in cSVD patients compared to controls. In the heterogeneous pattern of leakage, we observed a systematic increase of leakage in the perilesional white matter more distant to the WMH, which obscures differences in BBB leakage when assessing the NAWM as a whole. The WMH themselves were also found to have a greater tissue leakage volume in patients than controls.

We found much stronger leakage in the GM, compared to NAWM and WMH. This likely relates to the higher blood plasma fraction in the GM. The GM has greater microvascular density, and therefore a greater vessel surface area that potentially leaks.^26^ In the white matter, WMH show stronger leakage and less blood plasma volume compared to NAWM, which is especially pronounced in patients. This points to the pathological status of this tissue, being hypoperfused and having stronger BBB disruption. We additionally split the WMH up between periventricular and deep WMH. The finding that v_l_ was higher and v_p_ was lower in DWMH compared to PWMH underscores the value of assessing leakage around each WMH region individually. This suggests that DWMH are more pathologically altered than PWMH, with more leaking tissue and potential ischemia. However, it could also (partially) reflect inherent location-dependent microvascular differences or a contribution of peri-ependymal edema to the PWMH.

The approach of subdividing the NAWM into shells based on the distance from the WMH has revealed new relationships within our study cohort. Most prominently, higher K_i_ was found in perilesional NAWM of patients compared to controls, and it allowed us to pinpoint that this is most evident in the deep perilesional NAWM. Even though the lacunar stroke region was removed from ROI masks and patients were scanned >3 months after their stroke, sensitivity analyses pointed out that this effect was most evident in cSVD patients recruited due to having had a lacunar infarction. This points to either greater BBB permeability in this subgroup, or more preserved vascular surface area due to less microvascular rarefaction than the VCI group. The shell-based approach also showed a pattern of lower K_i_, v_l_, and v_p_ near WMH edges, increasing with distance from that edge (Figure 3). As expected, the ROI-averaged analyses confirmed that BBB leakage in the NAWM and the GM were positively related to age in our whole cohort, potentially contributing to age-related cognitive decline.^27^

When comparing our findings to previous literature, it becomes clear that detecting DCE-MRI derived BBB leakage in cSVD is challenging. While some studies find increased BBB leakage in cognitively impaired patients^23,28^, others do not find a significant leakage difference with controls^29^, or only see differences in v_l_, not in K_i_^30^, similar to our observations in the WMH. Likewise, some studies find positive relationships between BBB leakage and cSVD markers^12,31^, whereas others do not.^32,33^ These discrepancies underscore the subtle and heterogeneous nature of BBB leakage in cSVD. Our shell-based approach provided greater sensitivity for the detection of these subtle BBB leakage differences in evidently heterogeneous tissue.

The spatial heterogeneity analyses of the perilesional NAWM showed a plateau in K_i_ and v_l_ in both patients and controls after 4–6 mm. Patients and controls showed differences in terms of v_p_ pattern (Figure 3(e,f)). Patients show increased v_p_ farther from WMH. This could reflect a vasodilation response to compensate microvascular rarefaction in the perilesional NAWM, but which fails closer to the WMH and within WMH. Due to the growth of WMH over time, with the closest shells transitioning sooner than the farther ones, the shell distance could provide a proxy of the time course of this effect. This fits to the observation that the vasodilation capability (i.e. cerebrovascular reserve capacity) lessens over time (analogously closer to WMH) in patients with cSVD.^34,35^ In controls, where the WMH volume is only small, this effect is not seen, suggesting that controls have no or less rarefaction in the NAWM and preserved compensatory vasodilation relative to patients with cSVD in the WMH.

The observation that the leakage (both K_i_ and v_l_) was lower in the shells closest to the WMH and increased with distance is new and not shown before. This result was highly significant for shells surrounding WMH (both PWMH and DWMH), and can be observed in patients as well as controls. Previously, four studies have evaluated this distance relationship. The most recent study did not find any relationship between BBB leakage and distance from WMH in 44 sporadic cSVD and 32 cerebral autosomal dominant arteriopathy with subcortical infarcts and leukoencephalopathy (CADASIL) patients.^5^ However, the severity of disease in that sporadic cSVD cohort seems to be much lower, judging by WMH volume (mean (SD) of 12.0 mL (18.4) versus 25.3 mL (19.8) in the current study). Two other studies used an overlapping cohort of patients with cSVD and found increased leakage (both K_i_ and v_l_) closer to WMH.^11,13^ While their cohort is comparable to the patients evaluated in the current study, a different DCE-MRI methodology was used to evaluate BBB leakage with coarser spatial resolution (2 mm^3^ vs 1 mm^3^ voxels). That technique possibly suffers from partial volume effects at the WMH-NAWM border of the stronger WMH leakage and a less gadolinium-sensitive pulse sequence (Saturation Recovery vs Inversion Recovery). Finally, a study assessed BBB leakage in 201 patients with a mild ischemic stroke using linear mixed modeling of the signal enhancement slopes and found increased leakage closer to WMH.^12^ However, this population is substantially different from ours, as they included cortical stroke patients as well, who had much stronger BBB leakage in the deep GM and NAWM. The latter is also supported by the measured tissue contrast agent concentration curves, which were still rising on average after approximately 25 minutes, suggesting stronger leakage than clearance from the blood compartment, which is the other way around in our cohort.

Reduced BBB leakage near WMH may be explained by the well-documented decrease in cerebral blood flow close to (and inside) these lesions.^5,7,36^ Inside the WMH and in its periphery, K_i_ may be a non-negligible percentage of the local lower plasma flow (F_P_). In these areas, the assumption that K_i_ approximates the permeability-surface area product (PS) (i.e. permeability-limited) would be violated. In that case, a flow-limiting effect would be induced close to WMH, potentially explaining reduced mean K_i_ surrounding WMH edges, by reducing the leakage in the most affected areas (Figure A2).^37^ Mixed permeability and flow limitations could still allow for higher mean K_i_ in the less-perfused WMH. In the permeability-limited regime, reductions in vascular surface area due to microvascular rarefaction could alternatively explain the observed phenomenon. In the NAWM, microvascular rarefaction may be more severe close to WMH, leading to a lower local PS product, whereas severe BBB damage could increase the PS product inside the WMH. These interpretations highlight the need for more research into the complex interplay of permeability, vessel surface area, and blood flow surrounding (and inside) WMH.

Some strengths and limitations should be considered when interpreting this study. Among the strengths, this is the first study that included a control group when assessing perilesional leakage patterns, allowing us to compare spatial heterogeneity between patients with cSVD and controls. Additionally, as WMH in cSVD tend to grow into the surrounding NAWM over time, splitting the perilesional areas up by distance from the WMH may serve as a proxy for disease-stages. Finally, we employed a non-standard DCE-MRI methodology. This technique is more time-efficient due to only needing to sample one pre-contrast and two post-contrast time points and has a higher spatial resolution than DCE sequences used up to now, which is beneficial for our shell-based analyses. If partial volume effects from the (more leaky) WMH would be a concern, we would expect stronger perilesional BBB leakage, as seen in some other studies, whereas we found lower BBB leakage in the perilesional areas. A limitation may lie in the cross-sectional design, which does not allow us to draw concrete conclusions regarding disease progression or temporal causality of pathological alterations. Longitudinal studies would be beneficial to gain more insight into these dynamic processes. Moreover, the shell-based approach is subject to variability due to differing WMH volumes and locations between individuals, potentially confounding our results with inherent location-based differences related to tissue characteristics. Additionally, although we made a mutually exclusive distinction between distal PWMH and DWMH shells, some (distant) voxels are influenced by both types of WMH when shells near each other, which happens especially when WMH are large.

## Conclusion

Subdividing NAWM based on distance from WMH allowed us to demonstrate stronger perilesional leakage in cSVD patients than controls, mainly driven by the regions surrounding DWMH. This was mainly evident in the subgroup of cSVD patients that were recruited due to a lacunar stroke. Both patients with cSVD and controls showed lower leakage near WMH edges and the WMH themselves had a larger tissue leakage volume fraction in patients. The revealed spatial gradient of BBB leakage in patients with cSVD and controls may reflect local reductions in microvascular blood flow, smaller vessel surface area due to rarefaction, or a combination of both. These findings refine our understanding of neurovascular permeability resulting from interactions between BBB breakdown, microvascular blood flow, and rarefaction, especially in these perilesional areas.

## Supplemental Material

sj-pdf-1-jcb-10.1177_0271678X251364151 - Supplemental material for DCE-MRI reveals spatial pattern in heterogeneous blood-brain barrier leakage within white matter in cerebral small vessel disease Supplemental material, sj-pdf-1-jcb-10.1177_0271678X251364151 for DCE-MRI reveals spatial pattern in heterogeneous blood-brain barrier leakage within white matter in cerebral small vessel disease by Damon Verstappen, Joost J.A. de Jong, Paulien H.M Voorter, Maud van Dinther, Robert J van Oostenbrugge, Julie Staals, Jacobus F.A Jansen and Walter H Backes in Journal of Cerebral Blood Flow & Metabolism

## Data Availability

Data can be made available upon reasonable request.
