# Supplementary material for: DCE-MRI reveals spatial pattern in heterogeneous blood-brain barrier leakage within white matter in cerebral small vessel disease
Source: J Cereb Blood Flow Metab. 2025 Aug 4;45(11):2104–14. doi: 10.1177/0271678X251364151 (PMC12321814; doi:10.1177/0271678X251364151)
Supplement: sj-pdf-1-jcb-10.1177_0271678X251364151 - Supplemental material for DCE-MRI reveals spatial pattern in heterogeneous blood-brain barrier leakage within white matter in cerebral small vessel disease [file sj-pdf-1-jcb-10.1177_0271678X251364151.pdf]

Appendix

Age and sex effects in shell analyses

The shell analyses of K_i showed a significant relation with age in shells drawn from all WMH (p=0.021) and from DWMH (p=0.0053), similar to the NAWM as a whole. However, shells surrounding PWMH did not show a significant relation between K_i and age. We found increased v_l with age (p=0.048) across shells drawn around DWMH, but not from the PWMH or all WMH. Similar to the whole NAWM, v_p was also higher with higher age in shells around all WMH (p=0.027) and surrounding DWMH (p=0.007), but not surrounding PWMH. Sex was not related to K_i or v_l in any shells. However, v_p was higher in females in shells surrounding all WMH (p=0.040) and DWMH (p=0.015), but not PWMH.

Table A1: MRI acquisition protocol

	Double inversion-time gradient echo	First-pass DCE
Sequence type	Inversion Recovery Gradient Echo	3D Fast-Field Echo
Repetition time (ms)	8.3	5.3
Echo time (ms)	3.8	1.6
Inversion time 1 (ms)	650	N/A
Inversion time 2 (ms)	2100	N/A
Flip angle	6°	30°
Matrix size	240x240	128x128
Number of slices	180	21
Voxel size (mm)	1 x 1 x 1	2 x 2 x 6
k-space ordering	Center	Center
Frequency encoding direction	Inferior-Superior	Left-Right
Dynamic scans	N/A	70
Dynamic scan interval (s)	N/A	1.46
Contrast agent dose (mL/kg body weight)	N/A	0.1
Contrast agent injection rate (mL/s)	N/A	3
Total acquisition time (minutes: seconds)	4:36	2:09

Table A2: Linear mixed model results of K_i, v_l, and v_p in shells surrounding white matter hyperintensities (WMH).

Shells surrounding	Parameter	Coefficient (SE)	P-Value
**Leakage rate K_i ($\cdot 10^{-3}$ min^{-1})**			
All WMH	Shell distance	0.005 (0.001)	**<0.001**
	cSVD	0.048 (0.022)	**0.032**
	Age	0.003(0.001)	**0.021**
	Female sex	-0.015 (0.023)	0.509
Deep WMH	Shell distance	0.005 (0.001)	**<0.001**
	cSVD	0.065 (0.023)	**0.005**
	Age	0.004 (0.001)	**0.005**
	Female sex	0.001 (0.024)	0.954
Periventricular WMH	Shell distance	0.012 (0.001)	**<0.001**
	cSVD	0.041 (0.027)	0.125
	Age	0.002 (0.002)	0.194
	Female sex	-0.038 (0.028)	0.179
**v_l**			
All WMH	Shell distance	0.005 (0.000)	**<0.001**
	cSVD	0.032 (0.023)	0.16
	Age	0.002 (0.001)	0.09
	Female sex	-0.002 (0.024)	0.92
Deep WMH	Shell distance	0.004 (0.001)	**<0.001**
	cSVD	0.035 (0.025)	0.153
	Age	0.003 (0.001)	**0.048**
	Female sex	0.014 (0.026)	0.595
Periventricular WMH	Shell distance	0.009 (0.001)	**<0.001**
	cSVD	0.024 (0.024)	0.303
	Age	0.001 (0.001)	0.481
	Female sex	-0.021 (0.025)	0.386
**v_p**			
All WMH	Shell distance	0.000 (0.000)	**<0.001**
	cSVD	0.001 (0.001)	0.078
	Age	0.000 (0.000)	**0.027**
	Female sex	0.001 (0.001)	**0.04**
Deep WMH	Shell distance	0.000 (0.000)	**<0.001**
	cSVD	0.002 (0.001)	**0.032**
	Age	0.000 (0.000)	**<0.001**
	Female sex	0.002 (0.001)	**0.015**
Periventricular WMH	Shell distance	0.000 (0.000)	**<0.001**
	cSVD	0.001 (0.001)	0.125
	Age	0.000 (0.000)	0.22
	Female sex	0.001 (0.001)	0.172

Table A3: Linear mixed model results of K_i, v_l, and v_p in shells surrounding white matter hyperintensities (WMH), when the cerebral small vessel disease (cSVD) group is split up by inclusion criterion. Either due to vascular cognitive impairment or due to a lacunar infarction.

Shells surrounding	Parameter	Coefficient (SE)	P-Value
Leakage rate K_i ($\cdot 10^{-3}$ min^{-1})			
All WMH	Shell distance	0.005 (0.001)	**<0.001**
	VCI	0.022 (0.024)	0.348
	Lacune	0.111 (0.032)	**<0.001**
	Age	0.005 (0.001)	**0.001**
	Female sex	-0.017 (0.023)	0.442
Deep WMH	Shell distance	0.005 (0.001)	**<0.001**
	VCI	0.039 (0.025)	0.113
	Lacune	0.129 (0.033)	**<0.001**
	Age	0.006 (0.001)	**<0.001**
	Female sex	-0.001 (0.024)	0.982
Periventricular WMH	Shell distance	0.012 (0.001)	**<0.001**
	VCI	0.011 (0.028)	0.698
	Lacune	0.116 (0.038)	**0.003**
	Age	0.004 (0.002)	**0.013**
	Female sex	-0.040 (0.027)	0.141
v_l			
All WMH	Shell distance	0.005 (0.000)	**<0.001**
	VCI	0.009 (0.024)	0.706
	Lacune	0.088 (0.033)	**0.007**
	Age	0.004 (0.001)	**0.008**
	Female sex	-0.004 (0.023)	0.860
Deep WMH	Shell distance	0.004 (0.001)	**<0.001**
	VCI	0.010 (0.026)	0.689
	Lacune	0.096 (0.036)	**0.007**
	Age	0.005 (0.002)	**0.004**
	Female sex	0.012 (0.025)	0.637
Periventricular WMH	Shell distance	0.009 (0.001)	**<0.001**
	VCI	0.002 (0.025)	0.951
	Lacune	0.081 (0.034)	**0.018**
	Age	0.003 (0.002)	0.082
	Female sex	-0.023 (0.024)	0.339
v_p			
All WMH	Shell distance	0.000 (0.000)	**<0.001**
	VCI	0.001 (0.001)	0.202
	Lacune	0.002 (0.001)	0.073
	Age	0.000 (0.000)	**0.020**
	Female sex	0.001 (0.001)	**0.043**
Deep	Shell distance	0.000 (0.000)	**<0.001**
	VCI	0.001 (0.001)	0.105

WMH	Lacune	0.002 (0.001)	**0.040**
	Age	0.000 (0.000)	**0.006**
	Female sex	0.002 (0.001)	**0.016**
Periventricular WMH	Shell distance	0.000 (0.000)	**<0.001**
	VCI	0.001 (0.001)	0.340
	Lacune	0.002 (0.001)	0.063
	Age	0.000 (0.000)	0.105
	Female sex	0.001 (0.001)	0.183

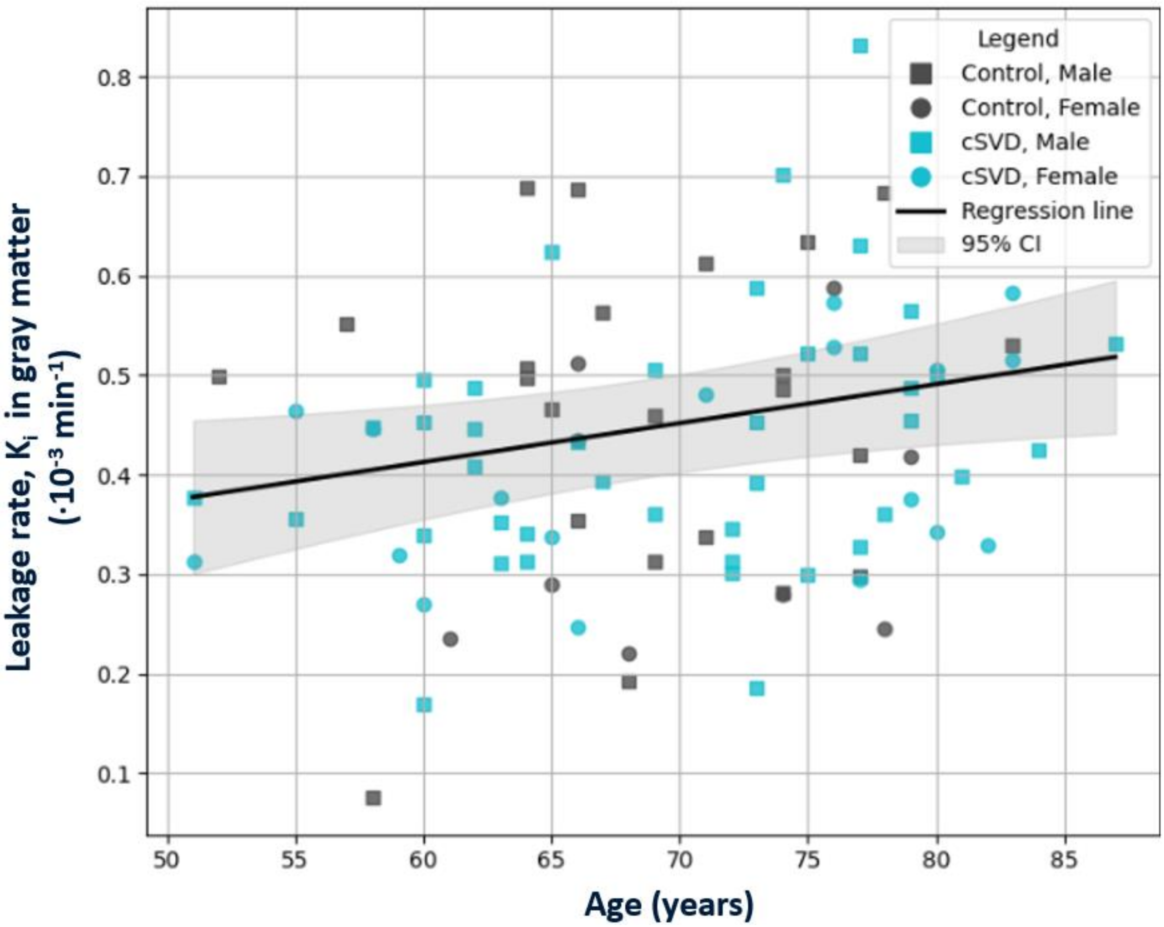

Figure A1: Scatterplot of the leakage rate, K_i in the gray matter and age. It includes a regression line and 95% confidence interval (CI) of that regression line for K_i in gray matter and age (for the example if the other

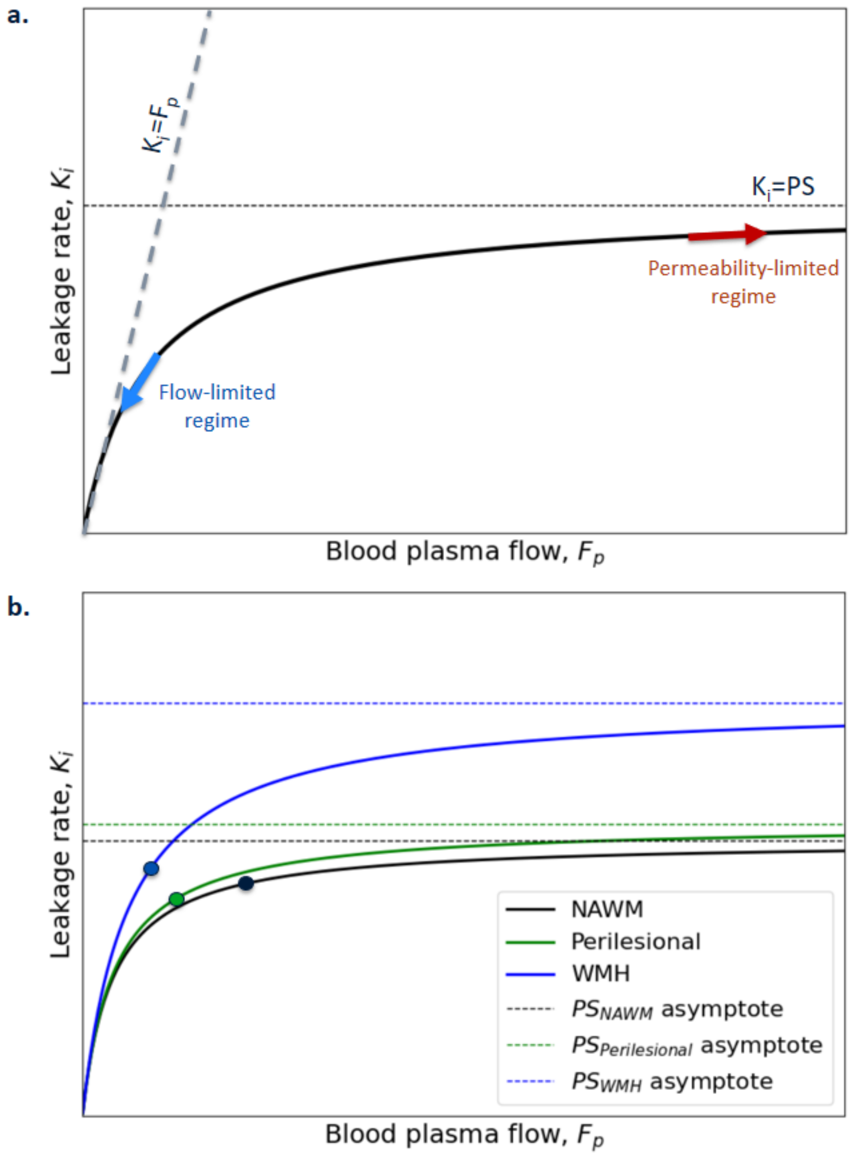

Figure A2: Conceptual illustration of flow- versus permeability-limited regimes where K_i denotes contrast agent extravasation to the extracellular extravascular space, F_p is the plasma flow, and PS is the permeability-surface product. For sufficiently high F_p, K_i approaches PS asymptotically.[1] For the current research we explain that the leakage in NAWM and WMH is in the intermediate range and therefore both flow and permeability limited.

References

1. Sourbron SP, Buckley DL. On the scope and interpretation of the Tofts models for DCE-MRI. Magn Reson Med. 2011;66(3):735-45.
